# Preparation, Modification, Food Application, and Health Effects of Protein and Peptide from Hemp (*Cannabis sativa* L.) Seed: A Review of the Recent Literature

**DOI:** 10.3390/foods14071149

**Published:** 2025-03-26

**Authors:** Xiaoqin Zhang, Wei Zhou, Xiaoli Qin, Chunsheng Hou, Xiushi Yang

**Affiliations:** 1 Institute of Bast Fiber Crops, Chinese Academy of Agricultural Sciences, Changsha 410205, China; zhangxiaoqin@caas.cn (X.Z.); zhouwei@caas.cn (W.Z.); qinxiaoli@caas.cn (X.Q.); 2 Changsha Technology Innovation Center for Plant Bioactive Ingredient Identification and Biosynthesis, Changsha 410205, China; 3 Hunan Provincial Key Laboratory of the Traditional Chinese Medicine Agricultural Biogenomics, Changsha 410205, China

**Keywords:** hemp seed protein, bioactive peptide, structure modification, food application, health effects

## Abstract

Hemp is a multiuse crop used for fiber, food, and medicinal purposes. The seed of hemp has attracted great attention as a good plant protein resource with remarkable nutritional and biological properties. However, the application of hemp seed protein (HSP) is limited due to its unsatisfactory functional properties. Physical, chemical, and biological technologies have been explored to modify the structure of HSP and improve its functionality. The investigation of the biological activity of HSP and its derived peptide to deal with intestinal, metabolic, and muscle concerns has broadened its utilization in healthy products. Therefore, the current review is performed to summarize the recent research progress on the novel extraction and modification of HSP, as well as the purification and identification of active peptide. The multi-functional multi-bioactive properties and adverse effects of HSP and peptide are also depicted to facilitate their potential applications in the food industry.

## 1. Introduction

Hemp (*Cannabis sativa* L.), a herbaceous plant belonging to the Cannabaceae family, originates from Central Asia and is cultivated for fiber, medicinal, and food purposes [1]. The Δ9-tetrahydrocannabinol (THC) content in hemp plants is generally below 0.3% or 0.2%, which is lower than *C. sativa var. Indica* (also called marijuana) [2,3,4]. Hemp is not only a sustainable and environment-friendly plant, requiring less water and pesticides during cultivation, but also an important cash crop, playing a key role in increasing farmers’ income [5,6].

As a traditional food and feed in China and India, hemp is able to generate considerable seed yield with high protein concentrations [7,8]. The popularity in the markets of hemp seed has been increased in recent decades based on its nutritional features. Owing to its balanced amino acid composition and being free of gluten and legume allergens, hemp seed protein (HSP) has been applied in several foods, including vegan protein bars and slimming protein smoothies, in European countries and Latin America [9,10]. Moreover, North America has also relieved its restriction on hemp cultivation and its protein utilization in food [11]. However, the application of HSP in food is still limited due to its unsatisfactory functional properties, such as poor solubility and emulsifying stability.

Efforts on using physicochemical and biological technologies to improve the structural features and functionalities of HSP were made in recent years [12,13]. Fermentation and enzymolysis were also utilized to release protein hydrolysates and peptides from HSP. In silico analysis has been performed to predict and identify biological peptides in an effective and quick way, which could potentialize their application in functional food [14,15]. There has been an elevated interest in the beneficial properties of hemp seed proteins and bioactive peptides, resulting in a series of published reviews [11,16,17,18,19]. However, research progress on the newly emerging structural modifications of HSP and peptides, as well as their applications in the food industry, are not well summarized.

Therefore, this review will describe the current knowledge of the modification methods and emerging food applications of hemp seed proteins and peptides. The newly discovered health effects of them will also be outlined to better promote the development of HSP-related functional products.

## 2. Extraction and Modification of HSP

### 2.1. Structural and Functional Properties of HSP

Hemp seed protein is mainly composed of edestin and albumin, with a β-sheet conformation as the predominant secondary structure [20]. HSP generally contains 11S and 7S globulin (around 65%~80%) and 2S albumin (around 20%~30%) [21]. The globulin is characterized by a large number of disulfide bonds, with a wider molecular weight distribution than albumin. The 11S globulin, as the major fraction of HSP, is responsible for its solubility and in vitro digestibility. The 7S globulin is a good candidate for manufacturing food gel [22]. The 2S albumin, distinguishing itself with a higher count of free sulfhydryl groups, might exist in a glycoprotein form to exhibit better solubility and foaming capacity [23,24]. The 2S albumin, accompanied with small-sized poly-peptides, possesses better interactions with water, resulting in higher foaming and emulsifying ability [22].

HSP contains all of the essential amino acids required for human health, regardless of the extraction or drying techniques employed, and their contents exceed the Food and Agriculture Organization’s recommended levels [11,23,25]. Arginine (3.64 g/100 g seed) is reported as the main amino acid in HSP, followed by glutamic acid (3.26 g/100 g seed), while lysine is the limiting one [26].

HSP has high surface hydrophobicity and low surface charge, inducing its low number of protein–water interactions and hindered solubility [11,27]. And HSP shows high propensity for aggregation and poor solubility. However, HSP possesses wet adhesion performance due to its low water solubility and water resistance. It can be utilized as an alternative source for plant-protein-based adhesives [28]. Moreover, considerable presence of unaggregated proteins can be rapidly absorbed to the air/water interface and air/oil interface, which will enhance its foaming and emulsifying attributes [24].

### 2.2. Extraction Method of HSP

Hemp seeds are always dehulled and defatted before protein extraction. Hemp seed proteins can be produced using different extraction methods. The most common methods are alkaline extraction coupled with isoelectric precipitation, salt extraction (also called micellization), and enzymatic extraction. Although alkaline extraction provides superior yield than extraction by water or aqueous NaCl, it produces unappealing and offensive brown (or greenish) color due to the oxidization of phenolic compounds under high alkalinity [29]. Conversely, salt-extracted HSPs are lighter in color and exhibit more albumin, oleosin, and sulfur amino acids [23].

Innovative technologies, in addition to the conventional methods above, have been employed for HSP extraction. Although salt extraction resulted in low protein yield, solubilization at 0.75 mol/L salt concentration coupled with ultrafiltration can still achieve 86.6% purity and 81.6% yield of protein [11]. High-pressure processing and ultrasound-assisted extraction, as green technologies, can improve the purity of extracted protein [30]. Furthermore, sub-/super-critical water, as a sustainable, safe, and economic alternative solvent, has been reported to be efficient in extracting hemp seed protein [31]. Additionally, the reversed micelle method will produce better hemp seed protein with higher solubility, foaming ability, and Arg/Lys ratio, which is assumed to be a solvent-recyclable, convenient, and easy to scale-up approach [32]. However, it is still necessary to make more attempts to further exploit green and effective extraction processes for this protein.

### 2.3. Modification Method of HSP

HSP generally show unsatisfactory functional properties such as poor overall solubility, emulsifying stability, and water-binding capacity due to its relatively high surface hydrophobicity and low surface charge [11]. Physicochemical and biological technologies have been employed to modify its conformation and functionality. Although several problems, including by-products, environment concerns, and resource consumption, are accompanied with the modification, the improved qualities of HSP can surely advance its development and adoption in plant-based foods. Table 1 presented the advantages and drawbacks of these modification methods.

#### 2.3.1. Physicochemical Technique

1. pH-shifting

pH-shifting is an easy and versatile chemical method to alter protein functionality. Low pH extraction could preserve the native state of protein, while the elevated pH would decrease the amount of amphiphilic protein [13]. The pH-shifting process would lead to the partial denaturation of protein, promoting losses of some tertiary structures, side-chain interactions, and exposure of sulfhydryl and hydrophobic cores [33]. Generally, pH-shifting alone is not sufficient enough for protein modification, which needs to be combined with other methods such as ball milling, ultrasonication, and high-pressure homogenization. Ball milling at pH 5 and lower could improve protein solubility by reducing its particle size and breaking the plant cell wall to facilitate protein collection [14]. Ultrasonication combined with pH-shifting could also lead to an increase in protein solubility, owing to the partial opening of the protein’s structure and facilitation of more protein–water interactions [13]. The protein obtained from pH-shifting and high-pressure homogenization demonstrates a greater unfolding structure by promoting its conformational transitions from β-sheet to α-helix and random coil [34]. Furthermore, treatments involving pH-shifting and manothermosonication (sonification under low hydrostatic pressure and mild temperature) could lead to a decrease in the α-helix, increase in random coil forms, enhancement in protein digestibility value, and improvement in solubility of HSP [33].

2. Protein–polyphenol interactions

Polyphenols can react with proteins in a non-covalent or covalent manner, altering the tertiary structure of proteins by exposing their buried hydrophobic sites to a hydrophilic and/or a polar microenvironment. Covalent interaction will change protein’s structure to a more unordered and unfolded structure [35]. Interactions between hemp seed globulin and hemp seed polyphenols lead to transitions in both the secondary and tertiary structures, as well as to improvements in solubility, emulsifying capacity, antioxidant activity, and digestibility [36,37]. Moreover, tannic acid with four phenolic hydroxyl groups on its catechol ring could be non-covalently combined with HSPs, which results in decreased α-helix content and a loose structure of protein complexes [38]. Additionally, the composite of chlorogenic acid and ultrasonically modified HSP is more stable than the original protein because of the unfolding of protein structure and the exposure of internal SH groups [39].

3. Maillard-driven conjugation

Maillard-driven conjugation, also called glycosylation, is a covalent grafting method to improve protein properties. The bonding of reducing sugars will enhance protein surface hydrophilicity, while sugar chain grafting can shift the isoelectric point to a lower pH. This method is generally time-consuming and leads to protein aggregation. However, ultrasonication- and manothermosonication-assisted Maillard conjugation could produce conjugates with increased solubility, foaming capacity, emulsion activity, and stability [40]. It is suggested that high-intensity ultrasound can modify the structure of proteins, thereby accelerating Maillard conjugation and improving solubility [41].

4. Blending other proteins

Recently, blending proteins from different origins has been considered to be a method to ameliorate the nutritional and functional properties of protein, which can adjust the amino acid profile and improve the techno-functional properties [42]. Sodium caseinate is a soluble animal protein in which may occur hydrophobic interactions, hydrogen bonding, and thiol–disulphide interchange reactions with hemp seed protein to improve its solubility. The solubility of hemp seed globulin could be increased by more than 80% by interacting with sodium caseinate after pH cycling [43]. Faba bean is an excellent foam stabilizer and exhibits complementary amino acid profiles with HSP, which is suitable for improving the foaming and nutritional properties of HSP [42]. Moreover, the blending of hemp protein hydrolysate with pea protein isolate could also provide a balance amino acid profile, as well as improving the digestibility and providing higher concentration of methionine and lysine [44]. These blends could not only meet the demand of vegetarians, but also tackle some limitations in plant protein in a simple and safe method.

#### 2.3.2. Bio-Technique

Germination and fermentation are modification processing methods that have been used over decades to improve the health potential of food materials [4]. Compared to other methods, they offer an alternative clean, safe, and economical biotechnological approach to modify protein functionality. Germination requires few inputs and produces minimal waste, which could effectively alter the functional properties of HSPs. After three days of germination at room temperature (∼22 °C) in the dark, hemp seed storage proteins will undergo significant degradation through endogenous enzymatic activity to produce hydrolyzed protein fragments [45]. The long-term (5 days) germinated HSPs exhibit improved solubility in neutral-to-slightly acidic pH and increased water holding capacity, foaming ability, and emulsifying properties. However, short-term (1 day) germination has negative impact on the solubility and foaming capacity of HSPs. In addition, malting for 3 germination days and kilning at 70 °C is able to heighten the protein content up to 9% [46].

Additionally, microbial fermentation could break proteins down into small peptides. The fermentation of hemp seeds with lactic acid bacteria results in improved protein extractability, solubility, and digestibility. It could not only increase the emulsifying capacity, foaming capacity, and the phytochemical amount of HSP, but also produce bioactive peptides and increase the product’s shelf-life [47]. Protein hydrolysis with *Bacillus subtilis* and *Lactiplantibacillus plantarum* both exhibit increased peptide content and promoted γ-aminobutyric acid accumulation [48]. The rich protease system of *Bacillus subtilis* could break down large protein molecules in the substrate into small polypeptides [49]. Hemp seed bran protein extract, hydrolyzed by alcalase after human distal colonic fermentation, could produce more beneficial organic fatty acids and probiotics [50]. Hemp seed fermented by *Aspergillus oryzae* can reduce the NO production in lipopolysaccharide-stimulated N9 microglial cells by downregulating inducible nitric oxide synthase expression, consequently decreasing inflammatory mediator and cytokine expression levels [51]. It should be noted that lacto-fermentation of HSPs could form undesirable compounds like biogenic amine [52].

## 3. Preparation and Identification of Active Peptides from HSP

### 3.1. Preparation of Peptides

The food applications of HSP are still limited due to its poor solubility. Peptides, with smaller molecular size and reduced spatial structure, possess better solubility and bioactivity than the original protein. Various methods, including enzymatic hydrolysis and chemical synthesis, have been used to produce peptides from HSPs [53,54]. The preparation methods, sequences, and bio-activities of these peptides are summarized in Table 2.

Enzymatic hydrolysis, including in silico and in vitro methods, is the most employed way for peptide preparation from HSP. Enzyme types (pepsin, trypsin, papain, Alcalase, Flavourzyme, etc.) and reaction conditions have played a great role in the protein recovery rate and peptide bioactivity. However, the generated bioactive peptide might possess some disadvantages, such as bitter taste, hygroscopicity, and low bioavailability [55]. Several measures could be taken to improve their functionality and bioactivity. Significant reductions in bitterness can be achieved using exo-proteinase digests such as Flavourzyme [66]. Additionally, the high Fischer ratio hemp peptides, which have more branch-chain amino acids and fewer aromatic amino acids, could be prepared by activated carbon adsorption, ultrafiltration, and gel filtration chromatography [56].

The chemical synthesis method for peptide mainly includes solid-phase synthesis and soluble-phase synthesis. The soluble-phase method is low-cost, but will produce plenty of intermediates and need purification at each step. The solid-phase synthesis overcomes this disadvantage, since it is based on the reaction of amino acids covered in insoluble compounds [67]. Meanwhile, solid-phase synthesis could dramatically simplify the synthetic production of peptides by allowing for the straightforward isolation of products with simple filtration. However, the main weaknesses of solid-phase synthesis are the overloading of raw materials and remarkable waste from the series of washing steps. A quick and efficient synthesis could be performed by using fluorenylmethoxycarbonyl (Fmoc) as the chemical group coating [68]. This protocol was successfully used to prepare HSP-derived peptides, which were characterized for different bioactivities [63]. In order to eliminate the waste from intensive washing, bulk evaporation at an elevated temperature could be combined during solid-phase peptide synthesis [69]. Furthermore, the large-scale synthesis of peptides can also be achieved using solid-phase synthesis. It has been reported that 1536 short peptides in good quantity are synthesized in 384-well plates by using this method [70]. Overall, the development of chemical synthesis has accelerated the exploration of active peptides and their modification.

### 3.2. Purification and Identification of Peptides

Initial protein hydrolysates are mixtures of peptides, non-degraded proteins, and free amino acids. Therefore, isolation and purification are required for further research. Size exclusion chromatography and membrane ultrafiltration are often applied to separate short-chain peptides [71]. Protein hydrolysates from hemp bran can be directly divided by membrane ultrafiltration into fractions of different molecular weights [57]. Additionally, ethanol graded precipitation can be used to obtain peptide with the best α-glucosidase inhibitory activity [72].

Mass spectrometry is widely used for the identification of peptides due to its high sensitivity, accuracy, and ease of operation. Matrix-assisted laser desorption ionization time-of-flight mass spectrometry, a rapid protein analysis technology, is applied to monitor protein modification during a bioprocess and determine food protein origin [73].

Traditional experimental approaches, including in vitro and in vivo assays, can precisely assess the bioactivity of peptides, although these methods are time-consuming and high-cost. Therefore, the use of in silico tools to identify bioactive peptides is increasingly growing [15]. High-throughput screening methods, including multi-omics, molecular docking, and machine learning technologies, with high mining efficiency, strong targeting ability, and low false-positive rates, are suitable for exploring biopeptides [14]. Moreover, in silico analysis combining bio-informatics tools and databases is effective in predicting and identifying peptide properties and sequences. However, it is not be an accurate enough tool to define bioactive peptides, and in vitro/in vivo analysis is still required [15]. The dipeptidyl peptidase IV (DPP-IV) and angiotensin I-converting enzyme (ACE) inhibitory active peptides from hemp seed protein isolate, screened using in silico analysis, could be further validated using an in vitro assay [74].

## 4. Application of HSPs in Foods

### 4.1. Improvements in Food Quality

HSP possesses high protein and well-balanced essential amino acids, which are highly suitable for food applications by increasing the nutritional value [75]. Moreover, HSP could offer improved functional and sensory properties due to their water and oil holding capacities, foaming ability, and emulsion capacity [20]. The typical application of HSPs in foods, including meat [76,77], bakery products [78,79,80], dairy products [81], and pasta [82,83], are summarized in Table 3.

### 4.2. Emerging Applications in Food

#### 4.2.1. Food Active Films

Plant proteins have gained great capacity for film formation, with advantages of safe edibility and rapid degradation under natural environments [84,85]. Bioplastics made from HSPs have higher hydrophobicity, less swelling, faster adsorption kinetics, lower surface tension, and a more irreversible adsorption of protein. Therefore, it is applicable in food packaging and coating technology [86]. Deccan hemp (*Hibiscus canabinus*) seed protein shows exceptional rheological properties, which might be suitable for making a coating material for fruit and vegetable preservation [87]. However, protein-based films generally exhibit poor mechanical and water vapor barrier properties, which limit their applications in food packaging.

The functional efficiency of food packaging could be enhanced by different preliminary treatments or adding various additives, including natural polymers, preservatives, and bioactive compounds. HSP film crosslinked by the microbial transglutaminase displays more homogeneous, smoother, resistant, but still flexible structure, and exhibits higher heat-sealing strength [88]. A composite active film produced from Deccan hemp seed protein, taro starch, and hemp leaf extract could improve the quality of grapes and prolong their shelf life [84]. Moreover, the edible films made from 50% sunnhemp protein, 50% potato starch, and 1% clove oil possess better mechanical strength and barrier properties [85].

The films could effectively protect food products inside the packages from the external environment, establishing a physical barrier to alter the internal atmosphere and preventing dehydration. Additionally, hemp seed proteins are deposited in small discrete storage organelles within plant cells, which are called HSP bodies. When pH shifted away from the pI, HSP bodies would swell, rupture, and eventually dissolute [21]. It is suggested that they are also potential pH-sensitive materials for monitoring food quality.

#### 4.2.2. Food Additives

HSP with amphiphilic properties can be used as an emulsifier [89]. Hemp seed protein isolate and globulin can be developed into emulsifiers in acidic food. Conversely, hemp seed albumin shows emulsifying properties in neutral and alkaline conditions because of its great solubility [24]. Meanwhile, the low solubility of HSP and the tendency to aggregate could partially affect its emulsifying ability. Therefore, several treatments were employed to assist in stabilizing the emulsions. For instance, gum arabic can react with HSP to thicken the oil–water interface [90]. Adding phospholipids into hemp protein could lead to the changes in its surface activity by better stabilizing the oleogel-in-water system [91]. In addition, physical treatments like ultrasound [39] and high-pressure microfluidization [90] could yield small droplets and narrow particle size distributions, thus increasing the stability of emulsions. Furthermore, HSP and its related peptides exhibit antiradical and antioxidant attributes for extending food shelf life, which can be the source of natural antioxidant ingredients [57,58].

#### 4.2.3. Encapsulating Materials of Bioactive Compounds

Protection is required during food processing and storage to maintain its quality, including its flavors, nutrients, and shelf life. Plant proteins could effectively encapsulate unstable and highly active compounds. Hemp seed globulin reveals potentiality to carry hydrophobic bioactive components, such as improving the solubility of Cannabisin A [37]. HSP can be used to coat hemp seed oil oleogel by forming oleogel-in-water emulsion, which shows higher oxidative stability, probably owing to the antioxidant components from HSP [92]. However, single material is generally not enough to enhance the functionality and stability of the encapsulated bioactive substances, and the combination of different wall materials is often performed. HSPs combined with different anionic polysaccharides could function as appropriate food-grade encapsulating materials or carrier matrixes and offer improved stability and functionality [93]. The nanoparticles developed with hemp seed globulin and alginate are capable for the encapsulation of Cannabisin A by demonstrating excellent resistance to high-ionic-strength environments and pepsin digestion [94]. Notably, hemp seed oil encapsulated by the hemp-seed-protein–alginate wall material maintains its antioxidant activities and oxidative stability well during storage [95]. Furthermore, hemp seed oil nano-emulsion could be stabilized by the compact complex of hemp seed protein isolate and alginate, even in high-salt environments [96].

## 5. Health Effects of HSPs and Peptides

### 5.1. Antioxidant and Anti-Inflammatory Effects

Radical scavenging capacity is an expression of antioxidant activity. Generally, radical scavenging activity is increased with protein hydrolysis degree, and higher activity is related to smaller-sized peptides [97]. HSPs hydrolysates possess DPPH and ABTS radical scavenging activity, and could improve the ferric reducing antioxidant power exerting an antioxidant effect in vitro [97,98]. The hydrolysates reduce the H_2_O_2_-induced oxidative stress in Caco-2 cells [55]. Furthermore, WVYY and PSLPA, extracted from HSPs, exert antioxidant and antibacterial effects and promote wound healing. The mechanism of these effects could be involved in the nuclear factor erythroid 2-related factor 2 (Nrf-2) signaling pathway [64]. Similarly, HSP-derived YGRDEISV and LDLVKPQ show antioxidant properties for their blockage of the entrance to myeloperoxidase active cavity [58]. Meanwhile, WVSPLAGRT and IGFLIIWV, hydrolyzed from hempseed protein, could reduce the level of H_2_O_2_-induced reactive oxygen species in HepG2 cells by modulating the Nrf-2 pathway [65].

Hemp protein hydrolysates exhibit high anti-inflammatory properties by modulating the release of several cytokines [59]. WVSPLAGRT and IGFLIIWV could also down-regulate the production of nitric oxide (NO) and nitric oxide synthase (iNOS), which are usually involved in inflammatory events [65]. Moreover, the two peptides are able to exert anti-inflammatory activity by regulating the pro-inflammatory and anti-inflammatory factors through the modulation of the nuclear factor kappa-B and iNOS pathways in HepG2 cells, respectively [60]. In addition, six chemically synthetized peptides (DDNPRRF, SRRFHLA, RNIFKGF, VREPVFSF, QADIFNPR, and SAERGFLY) originated from HSP exhibit anti-inflammatory activity by regulating the gene expression of TNF-α, IL-1β, IL-6, and IL-10 [63].

### 5.2. Benefits on Intestinal Health

An unhealthy lifestyle and dietary patterns contribute to higher prevalence of intestine disease like inflammatory bowel disease (IBD), which has become an increasing health concern worldwide. Numerous proteins have been filed to exhibit potential effects against IBD by repairing damaged intestinal tissue and enhancing immunity [99]. Hemp protein hydrolysates exert immunomodulatory properties in the human colorectal adenocarcinoma Caco-2 cell line, potentializing their beneficial effect for the intestinal epithelium [59]. Furthermore, the water-soluble proteins from hemp seed and their hydrolysates prove the lack of pro-inflammatory effects on U937 human leukemia cells and exhibit antioxidant and anti-inflammatory properties, highlighting their potential in alleviating gastrointestinal autoimmune diseases [61]. Hemp-seed-derived nanovesicles (containing 4906 µg protein in 1.25 g of fresh seed) could restore small intestine and colon injuries in mice induced by dextran sulfate sodium, which might be explored as a strategy to treat IBD [62]. Furthermore, in vitro colon fermentation evidences that hemp seed bran protein hydrolysate exhibits prebiotic potential by producing beneficial organic fatty acids and elevating beneficial bacteria for the intestines [50]. But there is still lack of information on the effect of hemp seed protein or peptides at the gastrointestinal level in vivo.

### 5.3. Relieving Metabolic Syndrome

#### 5.3.1. Antidiabetic Effect

Diabetes is a chronic metabolic disease characterized by hyperglycemia, which can easily cause a variety of complications involving cardiovascular, urinary, and nervous systems to threaten human health. Hemp-seed-derived inhibitors of DPP-IV demonstrates potential as novel therapeutics for diabetes. Sixteen DPP-IV inhibitory peptides are screened from HSP by molecular docking, and INS-1 cells experiments further validate their bioactivity on inhibiting cellular DPP-IV, enhancing glucagon-like peptide-1 (GLP-1) levels, and improving insulin secretion [100]. A tetrapeptide VAMP, mined by molecular docking and machine learning methods, could strongly inhibit DPP-IV (IC_50_ = 1.00 μM in vitro) and improve glucose metabolism in obese mice by increasing GLP-1 secretion and promoting the growth of gut microbial *Akkermansia muciniphila* [14]. Moreover, FNVDTE from hemp seed edestin and EAQPST from hemp seed vicilin also displayed therapeutic potential in managing diabetes through DPP-IV inhibition [101]. Additionally, enzymatic hydrolysate from non-dehulled hemp seed meal exhibits an inhibition effect on α-glucosidase activity and glucose transmembrane transport in Caco-2 cells [102].

#### 5.3.2. Antihypertension Effect

Hypertension is a chronic health issue described as blood pressure being consistently deviated from normal values. The effect of peptides on intervening or treating hypertension are mainly related to inhibiting ACE activity. Protein hydrolysate from hemp bran shows ACE inhibitory activity increasing with the molecular weight decreasing in peptide fractions [57]. A double-blind, randomized, crossover design trial recruiting 35 adults with mild hypertension was carried out concerning the antihypertension effect of HSPs and its peptides [103]. The results show that intake of both HSPs and peptides could decrease 24 h systolic and diastolic blood pressure, reduce plasma ACE activity, and increase renin and NO concentrations [104]. It is implied that the inclusion of hemp protein in the diet may potentially play a role in managing mild hypertension.

### 5.4. Mitigating Muscle Atrophy

Muscle atrophy is characterized by the loss of muscle mass and function, reducing the ability to carry out daily activities and extending recovery time from an illness. Intake of proteins could facilitate muscle protein synthesis and avoid muscle atrophy [105]. HSP peptides are found to mitigate muscle atrophy by modulating muscle protein degradation pathways in muscle atrophy mice induced by dexamethasone, as well as promoting skeletal muscle health to recover muscle mass, strength, and fiber size distribution [106]. Furthermore, HSPs hydrolysates could promote myotube formation and increase the expression of regulating factors for muscle protein synthesis. They could also protect C2C12 myotubes against dexamethasone-induced muscle atrophy by increasing the protein expressions of myogenic differentiation 1 and myosin-heavy chains [107]. Accordingly, HSPs and their peptides are potentially functional food ingredients that might promote muscle differentiation and prevent muscle atrophy.

### 5.5. Some Adverse Health Effects

Hemp seed proteins might show adverse effects, since the seed contains potential allergens. The five allergenic components (profilin, a non-specific lipid protein, oxygen-evolving enhancer protein 2 oxygen, the Bet v 1 homolog, and thaumatin-like protein) from hemp have been indexed in WHO International Union of Immunological Sciences allergen database, although most of them are not detected in hemp seed isolates [108]. However, an epicutaneous skin prick testing indicated that hemp seed exhibited potential allergenicity [109]. A previously reported allergenic protein from *Cannabis sativa* leaves, ribulose-1,5-bisphosphate carboxylase/oxygenase, was also characterized in hemp seed [110]. Furthermore, the 7S vicilin-like proteins and edestins in hemp seed were identified as potential allergens based on immunoblots and proteomics analyses [111]. A cross-reactivity between these hemp seed storage proteins and hazelnut was also revealed. Further studies are required to explore other cross-allergies associated with hemp seed proteins, as well as their other adverse health effects.

## 6. Conclusions and Future Perspectives

This review highlights the potential of hemp seed protein and peptides as emerging valuable bioactive ingredients to improve food quality and develop functional products. Furthermore, the extraction and modification methods of HSP, as well as the preparation and identification of active peptides, are summarized. Despite hemp seed protein being characterized as a good source of essential amino acids with multiple health benefits, the use of the cannabis plant has been stigmatized in most countries in the world due to its psychoactive effects. Accordingly, increasing science popularization and raising public acceptance about hemp is necessary to facilitate the promotion of related products. And more research is highly recommended to confirm the potential allergens in hempseed protein. Moreover, although in silico analysis can predict hemp seed active peptides properties and sequence as time- and cost-effective alternative tools, more in vitro analyses, animal tests, and human intervention trials are still required to better support their application in daily diets and as a functional food. In conclusion, this plant-based health protein offers an excellent opportunity to meet the demands of the food industry and benefit human wellness.

## Figures and Tables

**Table 1:** The benefits and drawbacks of modification methods for HSP.

Methods		Benefits	Drawbacks
Physicochemical technique	pH-shifting	Easy, versatile	Leading to the partial denaturation of protein, generally requiring combination with other methods
	Protein-polyphenol interactions	Improving functionality, digestibility, stability, and antioxidant activity	Not mentioned
	Maillard-driven conjugation	Enhancing surface hydrophilicity, lowering the isoelectric point	Time-consuming, leading to protein aggregation
	Blending other proteins	Improving amino acid profile, solubility, digestibility, etc.	Not mentioned
Bio-technique	Germination	Few inputs and minimal waste, improving solubility, foaming ability, emulsifying properties, etc.	Negative impact on the solubility and functionality by short-term germination
	Fermentation	Improving extractability, digestibility, product shelf-life, etc.	Forming undesirable compounds like biogenic amine by lacto-fermentation

**Table 2:** Preparation, sequence, bioactivity, and tested model of HSP-derived active peptides.

Preparation Method		Peptide Sequence	Bioactivity	Tested Model	Ref.
Enzymatic hydrolysis	Hydrolysis by different enzymes (trypsase, pepsase, papain, etc.)	VAMP	DPP-IV inhibitory, gut-microbial regulating	In silico, in vitro, in vivo	[14]
	Hydrolysis by digestive enzymes from *Aspergillus niger*, *Aspergillus oryzae*, and *Bacillus licheniformis*	QSFILG, ASVTKLG, ASVTKLG, KGVEIEG, VIEGKEG, etc.	Antioxidant	In vitro	[55]
	Hydrolysis by alkaline protease and papain	Not identified	Antioxidant, anti-fatigue	In vitro, in vivo	[56]
	Hydrolysis by Alcalase	LLY, LLR, IVY, LIY, IIY, etc.	Antioxidant, ACE-inhibitory	In vitro	[57]
	Hydrolysis by protamex	YGRDEISV, LDLVKPQ	Antioxidant	In vitro	[58]
	Sequential hydrolysis by Alcalase and Flavourzyme	MAEKEGFEWVSF, GLHLPSYTNTPQLVYIVK	Immunomodulatory	In silico, in vitro	[59]
	Hydrolysis by pepsin	WVSPLAGRT, IGFLIIWV	Anti-inflammatory	In vitro	[60]
	Simulated protease cleavage (trypsin, chymotrypsin, pepsin, etc.)	LPQNIPPL, YPYY, YPW, LPYPY, WWW, etc.	DPP-IV inhibitory	In silico, in vitro	[61]
	Putative hydrolysis by pepsin, trypsin, and chymotrypsin	FNVDTE, EAQPST	DPP-IV inhibitory	In silico	[62]
Chemical synthesis	Fmoc solid-phase synthesis	DDNPRRF, SRRFHLA, RNIFKGF, VREPVFSF, QADIFNPR, SAERGFLY	Immunomodulatory, anti-inflammatory	In silico, in vitro	[63]
	Synthesized by WELLPEP Co., Ltd. (Incheon, South Korea)	WVYY, PSLPA	Antioxidant, antibacterial, wound healing	In vitro	[64]
	Synthesized by the company GeneScript (Piscataway, NJ, American)	WVSPLAGRT, IGFLIIWV	Antioxidant	In vitro	[65]

**Table 3:** Typical applications of hemp seed protein in food.

Application	Examples	Quality Improvement	Ref.
Meat	Poultry roast: add hemp seed (8%), flour (0.2%), and oil (2%) to the recipes	Lower cooking losses; higher fiber, protein, aspartic acid, and arginine content	[76]
	Meat analogs: high moisture and high shear extrusion of hemp protein concentrates	Outstanding level of anisotropy; fibrous-like mesoscale structures	[77]
Bakery product	Cookie: replace 10% wheat flour with hemp protein preparation	Higher protein and threonine contents; delicate texture	[78]
	Bread: add 10% hemp press cake flour protein to the recipes	Higher protein content by maintaining similar textural properties	[79]
	Bread: replace 5% to 40% wheat flour with hemp seed cake flour	Higher protein and total phenol content; stronger DPPH radical scavenging activity	[80]
Dairy product	Cheese: replace 10% to 25% milk with hemp seed protein	Increased cheese yield; higher protein content; more balanced amino acid profile	[81]
Pasta	*Gnocchi* (potato-based fresh pasta): replace 5% to 20% wheat flour with hemp seed flour	Higher protein content; lower stickiness and cooking loss; good cooking resistance and firmness	[82]
	Rigatoni pasta: replace 5% to 20% wheat flour with hemp seed meal	Higher total phenolic content and antioxidant activity	[83]

## Data Availability

No new data were created or analyzed in this study. Data sharing is not applicable to this article.
